# The Centrosome: Conclusions and Perspectives

**DOI:** 10.3390/cells11233931

**Published:** 2022-12-05

**Authors:** Rustem E. Uzbekov, Tomer Avidor-Reiss

**Affiliations:** 1 Faculté de Médecine, Université de Tours, 10, Boulevard Tonnellé, 37032 Tours, France; 2 Faculty of Bioengineering and Bioinformatics, Moscow State University, Leninskye Gory 73, 119992 Moscow, Russia; 3 Department of Biological Sciences, University of Toledo, 3050 W. Towerview Blvd, Toledo, OH 43606, USA; 4 Department of Urology, College of Medicine and Life Sciences, University of Toledo, Toledo, OH 43607, USA

The centrosome consists of two centrioles surrounded by pericentriolar material. This unique subcellular structure has retained many features in the organisms of various taxonomic groups, from unicellular algae to mammals, spanning over one billion years of eukaryotic evolution. In addition to its most noticeable function, the organization of the microtubule system in mitosis and interphase, the centrosome performs many other cell functions. In particular, centrioles are the basis for the formation of sensing primary cilia, motile cilia, and flagella. Another principal function of centrosomes is the concentration of the regulatory proteins responsible for the cell’s progression throughout the cell cycle in a given location. Despite the existing exceptions, the functions of the centrosome are subject to general principles, which are discussed in this editorial. Below, we present nine papers in this collection, which address many of the critical aspects of centrosome biology.

A detailed analysis of the localization of various functionally active proteins recently identified their additional localization within the centrosome. Bergmann, together with the co-authors of the paper, shows that the SIRT4 protein is localized to the centrosome, although it was previously considered as exclusively mitochondrial [1].

Similarly, Mikulenkova, together with the paper’s co-authors, found that the nuclear protein NANOG/NANOGP8 is localized in the centrosome [2]. The above examples show that various regulatory pathways intersect in the centrosome; thus, regulatory proteins are concentrated in the centrosome. As noted in the review by Uzbekov and Avidor-Reiss, this location of the regulatory proteins is not random, since complex regulatory processes require several proteins to “meet in the cell” concurrently in one place [3]. Such a meeting at one specific location in the cell is more probable than the chance of meeting in the entire cellular volume or on the vast surfaces of the membrane components. The “point” localization of regulatory proteins allows the cell to synthesize them in significantly smaller quantities and quickly regulate or eliminate them as required, which greatly increases the speed and accuracy of the regulatory processes.

The centrosome core is the centriole pair. The centrioles are structures that consist of nine triplets of microtubules and are contained in the cells in strictly controlled quantities, specifically two per cell in G1 and four per cell during mitosis. As shown in the article by Sullenberger and co-authors, proliferating cells contain three structurally, biochemically, and functionally different types of centrioles: procentrioles, daughter centrioles, and mother centrioles [4]. These centrioles have differences in age and structure that are critical for the proper functioning of the centrosomes and cilia.

It has long been noted that the number of centrioles in cultured somatic cells, the most popular object of study among cell biologists, corresponds to the ploidy of the cells in each period of their cell cycle. Cells emerge from mitosis with a diploid set of chromosomes and two centrioles. During the interphase and up to the following mitosis, the number of chromosomes becomes tetraploid, and the number of centrioles increases to four (two centrioles and two procentrioles). The hypothesis of the non-randomness of such a correspondence is confirmed by the data on polyploid cells [5].

However, a study of the somatic cells of haploid male wasps showed that the number of centrioles in the haploid cells also changes in a manner identical to the diploid ones, i.e., there are two centrioles in a haploid cell after mitosis [6]. Thus, this correspondence in the number of centrioles and ploidy is nothing more than a coincidence. Similarly, haploid spermatids in insects and humans have two centrioles [7,8,9].

The more stable state of diploidy compared to haploidy in regard to DNA is explained by the possible compensation for damage to one of the chromosomes due to its sister chromosome. There are usually no accidents in evolution. The artificially obtained haploid cultures of vertebrate cells diploidize rapidly, partly due to the nondisjunction of the centrosomes during monopolar mitosis and, simultaneously, the death of another part of the haploid cells [10]. It is not clear why two centrioles are better than one for animal cells. This situation is probably due to the fact that these two centrioles are not identical [3,4] and perform different functions in the cell. 

Nevertheless, we can only state that, for centrioles, inheritance according to the 2/4/2 (two centrioles in G1/four centriole in mitosis/two centriole in the subsequent G1) formula turned out to be preferable to the 1/2/1 formula. This came to be evolutionarily fixed for all organisms and did not change, even for haploid organisms, such as the males of *Hymenoptera* [6]. 

Cilia are derivatives of centrioles and grow at their distal ends. Unlike centrioles, they do not contain MT triplets but rather doublets and lack the “C” microtubule (the external tubule in the triplet) [3]. Cilia are generally divided into two types: primary immobile cilia, whose functions are usually cellular sensation, and motile cilia, whose beating causes fluids to move across the cell surface, thus causing single cells to move. The two types of cilia also differ in their morphology. Primary cilia have the microtubule formula of 9 × 2 + 0, and they have nine peripheral doublets and lack central microtubules. Motile cilia have the formula of 9 × 2 + 2, and in addition to nine peripheral doublets of microtubules, they have two more single central microtubules. Sperm flagella are morphologically and functionally similar to motile cilia. 

Based on the data previously obtained [11,12] from the study of the spermatids and spermatozoa of vertebrates, the review by Uzbekov and Avidor-Reiss put forward a hypothesis asserting a fundamental difference in the foundations for the growth of primary and mobile cilia [3]. Primary cilia always grow on a more mature mother centriole that is more than one cell cycle old [3,4].

In contrast, motile cilia in the ciliary epithelium are formed on centrioles that appear in the same cell cycle as the cilia [13,14]. Therefore, these centrioles are, by definition, daughter centrioles. 

Vertebrate spermatids are unique and simultaneously have flagellum- and cilium-like structures on both centrioles. More precisely, the motile flagellum is formed on the daughter centriole [11]. On the more mature mother centriole, an analog of the primary cilium grows: the centriolar adjunct, which, like the primary cilium homolog structure, does not have central microtubules and cannot move [11,12].

The article by Arslankhan and co-authors is devoted to the study of the centrosome/primary cilium complex [15]. Among these complexes, the authors also included a related structure named the centriolar satellites. These are electron-dense formations that are often found near the centrosome and, as has been shown, are the sites of concentration of many important proteins involved in cell regulatory processes [15]. The authors note that the application of labeling methods based on the proximity to the centrosome/cilium complex generated maps of the spatial and temporal interactions of its components. In this way, the authors provided key insights into these issues and an extensive review of the research in this area, indicating the proteins and protein complexes identified through these methods. The spatial and temporal interactomes generated among a wide range of centrosomes, cilia, and satellite proteins have been instrumental in revealing new actors and mechanisms determining the ways in which the centrosome/ciliary complex is assembled, maintained, and dynamically changed [15]. In their article, the authors proposed techniques and methods for further research on the interactions of protein complexes in the centrosome/primary cilium/centriolar satellite system. They note the important role of these studies, including their medical importance, in terms of the treatment and diagnosis of ciliopathies of various etiologies.

In most cells, the centrosome is the dominant microtubule-organizing center. It contains microtubule nucleation proteins, mainly gamma-tubulin, which forms two types of complexes with other proteins [16]. However, in some cases, cells have non-centrosomal microtubule organization centers. Riparbelli and co-authors showed that Drosophila spermatids have an additional focus on γ-tubulin, localized at the anterior pole of the nucleus, in line with the apical end of the perinuclear microtubules running through the interior of the dense complex [17].

The review by Burakov and Nadezhdina [18] summarized the extensive data on the centrosome’s position in various cell types and the cellular mechanisms of the centrosomes [19,20]. The roles of the microtubules, systems of actin filaments, and intracellular motors in these processes were demonstrated. The authors presented evidence suggesting that the central location of the centrosomes is mainly due to the pulling forces of microtubules developed by dynein, located in the cell cortex and intracellular vesicles. Repulsive forces originating from the growing microtubules and the interaction between actin filaments and myosin also play contributory roles.

Centrioles are the structural basis of the centrosome due to their unparalleled stability, which provides the basis for the accumulation, formation, and physical interactions of protein complexes. What happens if the centrioles in the cell disappear? A method for such an artificial removal of the centrioles using centrinone, a reversible inhibitor of Polo-like kinase 4 (Plk4), has already been described [21]. However, there also exists a more “natural” model for studying cells without centrioles: the Drosophila melanogaster cell line 1182-4, which constitutively lacks centrioles [22]. This cell line was derived from homozygous haploid embryos laid by females for maternal haploid mutation [23]. Surprisingly, the complete absence of centrioles did not lead to catastrophic consequences in the life of these cells. Unlike PLK4-deficient cells without centrioles, the 1182-4 line lost the ability to form centrioles de novo. These data support the old hypothesis that centrioles are passengers at the poles of cell division, and their main function is the formation of cilia and flagella [24,25]. 

Often, the exceptions to rules allow us to better understand the rules themselves. One such exception in centrosomal biology is the centriole structure observed in some *Hexapoda* groups [26]. An interesting remark by Riparbelli and co-authors suggests that this group of organisms, which are the most numerous in the animal world and live in different habitats, represent a kind of living laboratory. Indeed, throughout their long evolution, insects have accumulated many mutations that have affected all the parts of these organisms. Even centrioles, which have retained their fundamental structure throughout the process of evolution from ancient flagellates to vertebrates, have significant structural variations in insects. The authors of this publication give examples of such deviations in the structure of centrioles from the canonical form. Such deviations include centrioles consisting of doublets, and this type of centriole is typical for somatic cells. The discovery of groups of *Hexapoda* with centrioles of an unusual structure that deviates from the generally accepted nine-arm symmetry raises the question of the roles of the cartwheel and the Sas6 protein in the assembly of centrioles, as described in the case of model organisms. To the results of this work on the diversity of centriole shapes [26], we can add another type of deviation, which we observed in wasps: centrioles without microtubules [27]. At the same time, these forms retained a ninth-order symmetry and contained nine prongs, which, together, formed a cogwheel structure. We identified this structure in the somatic cells of the larvae and at the base of the sperm flagella. In the spermatids, at the onset of the formation of the sperm flagella, the centrioles, as observed in *Drosophila* flies, contained nine triplets of microtubules [28]. A similar situation involving different structures of the centrioles in different cell types of the same organism was also described earlier in the case of the worm *Caenorhabditis elegans*. In this case, the centrioles contained nine singlets or nine triplets of microtubules [29,30].

The centrosome was discovered almost 150 years ago [31,32,33]. Over these many years of research, its role has changed from a cytoplasmic granule to a central complex of intracellular regulation [34]. However, some aspects of the formation and functions of the centrosome are still far from being fully understood. Here, we note only two such aspects.

Regarding the fundamental of the onset of centriole duplication in the cell cycle, researchers have not yet agreed on a unanimous opinion. Many reviews, including the work presented in this Special Issue [4], note that the onset of duplication occurs after the beginning or simultaneously with the start of DNA replication, that is, already in the S phase of the cell cycle. Based on the analysis of the indirect data from earlier works [35,36,37] and his data [38,39], Uzbekov argued that centriole duplication begins earlier, in the second half of the G1 phase, after the cell has already passed the restriction point. Different points of view may be associated with varying cell types and objects of study, and the sequence of these events in the cell may depend on some external conditions.

The second important and unresolved issue is the mechanism of centriole formation. Centrosome formation in cultured cells is already well understood. However, the means through which the centriolar apparatus of somatic cells is formed in the process of early embryogenesis from one typical proximal centriole and one atypical distal centriole, which the spermatozoon incorporates into the centriole-free oocyte, are still unknown [40]. Our upcoming article, which readers will be able to see in the near future, is devoted to providing a solution to this issue [41].
